# Development and validation of a machine learning model for on-site prediction of coronary heart disease in high-risk adults using clinical data

**DOI:** 10.1371/journal.pone.0334881

**Published:** 2025-11-13

**Authors:** Liwen Mo, Hua Lin, Chengxuan Li, Lifei Yu, Decheng Lu

**Affiliations:** 1 Health care center, the Second Affiliated Hospital of Guangxi Medical University, Nanning, Guangxi, China; 2 Department of Cardiology, the Second Affiliated Hospital of Guangxi Medical University, Nanning, Guangxi, China; 3 Guangxi Medical University, Nanning, Guangxi, China; Kurume University School of Medicine, JAPAN

## Abstract

**Background:**

Risk of coronary heart disease (CHD) in a specific period of years can be assessed using scores calculated by models, such as pooled cohort equations (PCEs) and Framingham Risk Score. However, there are few studies on on-site estimation of CHD risk quantitatively with score calculation as auxiliary diagnosis. Nowadays, researchers introduce new technologies, such as machine learning, as effective CHD risk prediction models, but these models still need to be validated using real clinical data before promoting their use in real clinical settings.

**Objective:**

The aim of this study is to predict CHD risk for high-risk population only using clinical data consisting of vital traits, lab measurement, diagnosis, medical device testing and medications. The prediction model can serve as an on-site quantitative indicator for the CHD risk of potential patients before diagnosis using coronary arteriography.

**Methods:**

This work is designed as a retrospective study of a hospital-based cohort (The Second Affiliated Hospital of Guangxi Medical University), comprising 20,821 patients with CHD and 9,796 controls from 2017 to 2024. A two-layer machine learning model (TLML) is developed on the prediction results of the random forest and the gradient boosting decision tree to combine the merits of both models. The models were trained and validated with the clinical data in the cohort.

**Results:**

The TLML presented in this study can have a good accuracy (0.79, 95% CI 0.79–0.80), sensitivity (0.79, 95% CI 0.79–0.80) and specificity (0.79, 95% CI 0.79–0.79) for on-site CHD prediction. Compared with the PCEs (accuracy = 0.59, sensitivity = 0.58 and specificity = 0.60), the TLML shows remarkably better on-site CHD prediction performance. Predictor importance analysis results show that age, diabetes, antihypertensive medications, total bilirubin, hypertension, obstructive sleep apnea-hypopnea syndrome, red cell count, hemoglobin, cystatin C, retinol-binding protein, gender and low-density lipoprotein cholesterol level are the most important variables for on-site CHD prediction. All the features mentioned were reported to have relationship with CHD on some levels in previous studies. A reduced complexity model is also presented to provide decent CHD prediction with only 20 predictors to increase model practicality, achieving a prediction accuracy of 0.73.

**Conclusions:**

The machine learning models presented in this study have the potential to become auxiliary on-site diagnostics tool of CHD because of its capability for accurate prediction and easy availability of all the required prediction variables.

## 1 Introduction

According to epidemiology studies, atherosclerotic cardiovascular disease (ASCVD) remains one of the noninfectious chronic diseases with the highest morbidity and mortality, with coronary heart disease (CHD) considered the top. The number of patients suffering from CHD in China has reached 11.39 million in 2022, accounting for around 3.45% among all ASCVD patients (330 million). To make matters worse, there is an obvious increasing trend of mortality in both rural and urban areas, reaching 135.88 and 126.91 per million population respectively [1].

Prediction models are developed to mitigate the ASCVD risk and control its impact (CHD included). The models enable early identification of individuals to better provide therapeutic strategies. The pooled cohort equations (PCEs) have been included in the American College of Cardiology/American Heart Association guideline as ASCVD risk prediction tool in the next 10 years by using the scores calculated by preset equations [2]. The effectiveness of the PCEs in risk assessment is further researched in other studies [3]. Earlier than the PCEs, Framingham 10-year general CHD risk calculation was recommended for the National Cholesterol Education Program Adult Treatment Panel III guidelines for high blood cholesterol in adults [4,5]. Other calculation models, such as SCORE2 [6], the systematic coronary risk evaluation model in Europe [7], Chinese oriented ASCVD prediction model (China-Par) [8], have been developed to apply ASCVD risk prediction on different population. The advantage of these risk assessment tools is the capability of using a few predictors to determine CHD risk of individuals qualitatively in the next period. However, there are also some limitations of these models. First, the relationship between CHD risk and clinical features should be quite complex, but the features as input for these models are quite limited. For example, researchers reported that the PCEs score inaccurately estimates the risk [9] and introduces biases in certain populations [10]. Second, the applicability of the model could be difficult in a large population. For example, the PCEs input race selection in the open-source calculator [11] only contains “White or Other” and “African American” options. So, inclusion of all other diverse races into “Other” makes the PCEs’ prediction accuracy questionable. These limitations make individuals with CHD risk not well covered by risk prediction in reality.

Recently, machine learning models are applied for 5-year or 10-year risk of CHD on the basis of electronic health records (EHRs), resulting in a good prediction for CHD risk with an area under the receiver operating characteristic curve (AUROC) of 0.95 (sensitivity of 0.94 and specificity of 0.82) [12]. Another machine learning model was proposed [13] for 1-year before diagnosis CHD prediction, and the result of which was compared with the PCEs to have a 20% increased discrimination and 34.4% increased reclassification in a subgroup with low CHD risk. The high prediction accuracy and inclusion of a large number of clinical features make the machine learning model a reliable candidate for CHD risk prediction.

Most of the machine learning models for CHD risk prediction focus on the prediction of future risk for individuals at hospital discharge. However, there are few studies on the prediction of on-site CHD risk with the vital traits and lab measurement upon admission from realistic hospital operations, which is also quite meaningful. The current diagnostics for CHD depend on coronary angiography, which could be expensive, time-consuming and traumatic. Machine learning models with accurate CHD prediction can also reduce the number of unnecessary coronary angiography to improve hospital operation efficiency and serve as a quantitative indicator for CHD diagnosis. Early diagnostics of CHD with models can contribute to intervention of coronary artery lesions, which decreases the incidence of acute myocardial infarction, death caused by ASCVD and sudden cardiac death.

In this work, a two-layer machine learning model (TLML) was developed by applying neutral network (NN) on the prediction scores of two well-studied machine learning models, random forest (RF) and gradient boosting decision tree (GBDT), to quantitatively calculate the on-site CHD risk with clinical data obtained by lab measurements, medication usage, medical device testing, personal and family histories and vital traits. This TLML can serve as an auxiliary diagnosis tool before coronary angiography. A reduced complexity model (RCM) was developed by selecting top 20 predictors from TLML model. We compared the results from machine learning models and the PCEs to illustrate the prediction performance.

## 2 Methods

### 2.1 Sample cohort

EHRs (personal information excluded) of the study cohort from the Second Affiliated Hospital of Guangxi Medical University, Guangxi, China, were extracted and analyzed to develop a prediction model for on-site CHD risk. All methods employed in this study were in accordance with applicable ethical guidelines and regulations. The data used in this study was approved by the Medical Ethics Committee of the Second Affiliated Hospital of Guangxi Medical University (approval number: 2024-KY(0044)), and data analysis was performed following the standard methods published in previous research or using commercially available analysis software that adheres to established protocols and industry standards. Written informed consent was obtained from all individual participants included in the study. A total number of 30,617 individuals were included in this study, among whom 20,821 patients had CHD, and 9,796 patients were admitted to the hospital with other diseases (controls). EHRs underwent a pre-treatment process before separating into the training and validation cohorts for further development and validation of the prediction models.

### 2.2 Clinical features of electronic health records

EHRs from the hospital included categorical, continuous, and textual data within the time window of January 2017 to April 2024 (data accessed on May 21, 2024). Only EHRs obtained immediately before hospital admission were used as effective data. Clinical features included routine blood tests, liver function tests, renal function tests, lipid panel analysis, thyroid function tests, and erythrocyte sedimentation rates. Data on medical device testing encompassed electrocardiography, transthoracic echocardiography, and color Doppler echocardiography of the carotid artery. Categorical features included disease classification, medications, race, and residence. The clinical features in text format were converted into categorical format by extracting keywords from the text for the resultant categorical data to be used for prediction model development. Continuous clinical features were also converted to categorical data by determining whether they conformed to the hospital’s practical guide in order for two kinds of clinical data—namely, all-categorical data and categorical/continuous mixture data—to be used separately for prediction model development.

**Fig 1:**
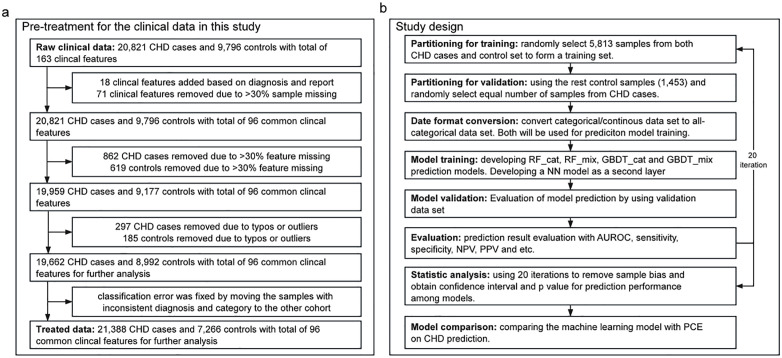
The data treatment and study design. a. clinical raw data treatment protocol; b. the design of prediction model development. CHD: coronary heart disease; RF: random forest model; GBDT: gradient boosting decision tree; cat: model trained with all-categorical data; mix = model trained with mixture of numerical and categorical data; AUROC: area under receiver operating characteristic curve; PPV: positive predictive value; NPV: negative predictive value.

Features with >30% of missing values in all individuals were discarded in feature engineering to improve data integrity and model training quality. Additionally, individuals with >30% missing features were discarded from the EHRs (Fig 1a). The remaining missing values were imputed with a surrogate using an RF based algorithm. Clinical records with basic features outside the regular scope (e.g., individuals with height outside of 100–220 cm, weight outside of 30–200 kg, or systolic blood pressure (SBP) outside of 70–200 mmHg) were also removed. These outliers were believed to be the result of typos in the hospital’s daily services. Finally, classification errors due to typos in the disease diagnosis codes were fixed by moving the samples with inconsistent diagnosis codes and medical reports to the group indicated by the medical reports.

The detailed pre-treatment procedure for EHRs (Fig 1a) comprised 3 steps: data conversion, filtering and randomizing. The final data consisted of 96 clinical features, including 9 vital traits, 4 personal histories, 4 family histories, 1 medicine intake, 63 laboratory results, and 15 comorbidities, which were included in the training and validation of the machine learning models in the following analysis steps (details of the typical features are shown in Table 1). No other prior selection was performed to preserve potentially informative predictors and minimize selection bias.

**Table 1:** Typical baseline characteristics of patients with CHD and controls by mean value and standard deviation or percentage (in bracket). CHD: coronary heart disease.

Characteristics	Whole cohort (N = 28,654)	CHD patients (N = 21,388)	Controls (N = 7,266)
Mean age	62.3(13.4)	65.1(11.1)	54.2(16.1)
**Sex**			
Male	18,149(63.3%)	14,429(67.5%)	3,720(51.2%)
Female	10,505 (36.7%)	6,959(32.5%)	3,546(48.8%)
**Self-reported ethnicity**			
Han	18,100(63.2%)	13,725(64.2%)	4,375(60.2%)
Zhuang	9,618(33.6%)	7,013(32.8%)	2,605(35.9%)
Other	936(3.3%)	650(3.0%)	286(3.9%)
Ever smoking	8,844(30.9%)	7,332(34.3%)	1,512(20.8%)
Ever drinking	7,941(27.7%)	6,315(29.5%)	1,626(22.4%)
**Median vitals (typical vital traits)**			
Height, cm	162.1(8.3)	162.2(8.3)	161.8(8.4)
Weight, kg	63.0(12.1)	63.2(11.7)	62.4(12.9)
Body mass index	23.9(3.7)	24.0(3.6)	23.8(3.8)
Systolic blood pressure, mmHg	131.8(20.9)	132.8(20.7)	128.6(21.4)
Diastolic blood pressure, mmHg	77.0(13.2)	77.0(12.9)	77.3(14.1)
Temperature, °C	36.5(0.3)	36.5(0.3)	36.5(0.3)
Pulse, sec^-1^	80.5(15.4)	79.9(14.4)	82.7(18.2)
**Median lab measurement (typical features)**			
Low density lipoprotein cholesterol, mmol/L	2.6(1.0)	2.5(1.0)	2.8(0.9)
High density lipoprotein cholesterol, mmol/L	1.1(0.3)	1.1(0.3)	1.2(0.3)
Total cholesterol, mmol/L	4.1(1.2)	4.0(1.2)	4.3(1.1)
Lipoproteins (a), g/L	62.0(78.6)	68.3(82.5)	48.8(64.5)
Apolipoprotein A1, g/L	1.2(0.3)	1.2(0.3)	1.3(0.3)
Triglycerides, mmol/L	1.6(1.4)	1.6(1.4)	1.5(1.2)
Glucose, mmol/L	5.6(2.0)	5.9(2.3)	5.0(1.3)
Hemoglobin, g/L	126.0(21.7)	124.1(22.4)	131.4(18.7)
Lactate dehydrogenase, U/L	215.2(185.0)	220.2(200.3)	200.3(127.9)
Homocysteine, μmol/L	13.9(9.8)	14.4(10.4)	12.7(7.9)
**Medical device testing**			
ST-T change	3,723(13.0%)	3,646(17.0%)	77(1.1%)
Left ventricular hypertrophy	1,785(6.2%)	1,651(7.7%)	134(1.8%)
Functions of contraction weakening	1,585(5.5%)	1,079(5.5%)	506(5.6%)
Arteriosclerosis	1,838(6.4%)	1,437(6.7%)	401(5.5%)
**Comorbidities**			
Hypercholesterolemia	4,740(16.5%)	3,424(16.0%)	1,316(18.1%)
Hypertension	17,458(60.9)	14,416(31.9%)	3,042(45.2%)
Pre-diabetes or Diabetes	8,549(29.8%)	7714(36.1%)	833(11.5%)
Fatty liver	3,701(12.9%)	2,654(13.5%)	1,047(11.6%)
Hyperuricemia	3,042(10.6%)	2,328(10.9%)	714(9.8%)
**Medicine taking**			
Hypertension depressor	21,203(74.0%)	17,015(79.6%)	4,188(57.6%)

### 2.3 Study design

In this retrospective cohort study, we developed an innovative TLML prediction machine learning model by introducing a second-layer NN model into the prediction results of the RF and GBDT models. The RF and GBDT models were first trained using all categorical data and categorical/number mixture data respectively. Four prediction models were generated, with two for the RF model (RF_cat model and RF_mix model) and two for the GBDT model (GBDT_cat model and GBDT_mix model). The prediction scores from these four models were then used for the NN training as a second-layer machine learning model layer. The five models were then evaluated for their predictive performance using the validation cohort. The model development and evaluation process was repeated for 20 iterations (Fig 1b) to minimize sampling bias. During each iteration, the training set was first built by randomly selecting 7,547 CHD cases and 7,547 controls (Fig 1b), whereas the validation set was constructed by randomly selecting 1,479 CHD cases and 1,479 controls without sample replacement. Finally, a Brier score optimization was applied to generate a quantitative threshold for low- and high-risk classifications. The hyperparameters of NN, RF, and GBDT were optimized separately with internal 10-fold cross-validation using only with the training set. The same model development process was conducted with both training and validation dataset size scaled from 30% to 100% (original study design) to evaluate the influence of sample size on the predictive performance.

In addition to the machine learning models, the PCEs were also introduced in this study as a benchmark of prediction performance, though they were originally designed for 10-year risk prediction. The PCEs were applied to all applicable individuals from the total cohort (with no missing values for any item required by the PCEs calculator). The items required by the PCEs were gender, age, race, total cholesterol, high-density lipoprotein (HDL) cholesterol, SBP, receiving treatment for high blood pressure, diabetes and smoking status. Owing to the data integrity, only 11,170 samples were extracted from the total cohort for PCEs calculation. The PCEs results of the validation set from the 20 iterations were analyzed separately and used for performance comparison, excluding those that could not meet data input requirement of the PCEs calculator.

Predictor importance analysis was performed by permutating out-of-bag observations to identify the most relevant clinical features in CHD prediction. The 12 most relevant clinical features were subsequently discussed for their relationship with CHD based on the results of previous studies.

The top 20 most important clinical features were extracted as the inputs for the RCM, which is a simplified version of the TLML. The RCM can be more practical than the TLML because of the fewer clinical features required, but it had acceptable accuracy. The PCEs and RCM were also compared in terms of prediction performance.

### 2.3 Statistics analysis

The cohort baseline was evaluated by applying mean value, percentage and standard deviation calculation. The accuracy for prediction outcomes of the model present in this study was assessed via the area under the receiver operating characteristic curve, F-scores, and Brier scores. Direct indicators such as accuracy, specificity, sensitivity, negative predictive value (NPV) and positive predictive value (PPV) were also used as detailed predictive measures. The 95% confidence interval (CI) was calculated across 20 iterations as an indicator of prediction consistency. The *t*-test and *p*-value were applied to evaluate the difference in prediction performance among the models, with a significance level setting at 0.05. The prediction score from the TLML was used as a quantitative indicator of on-site CHD risk, and a threshold for classifying low and high risks was obtained by optimizing the Brier scores.

Pretreatment of the raw data, model development, validation, and statistical analysis were performed via in-house coding using data treatment functions and internal machine learning libraries in MATLAB 2024a. The PCEs evaluation of the 10-year CHD risk was performed using an online open-source calculator [11].

## 3 Results and discussion

### 3.1 Baseline characteristics of the cohort

This study included 30,617 cases (raw data), comprising 20,821 CHD cases and 9,796 controls with 163 clinical features. The raw data underwent pretreatment before being used for subsequent analyses (Fig 1a). The raw data contained clinical information categorical, in continuous and text formats, which were first converted into features that could be used for machine learning model training. After data conversion, 18 additional clinical features were generated, which were filtered for sample integrity (>30% sample missing), resulting in the removal of 71 clinical features (Fig 1a). Subsequently, 862 CHD cases and 619 controls were excluded owing to >30% missing values. Finally, individuals with abnormal height, weight, and blood pressure were removed from the samples as outliers or typos (Fig 1a). The remaining dataset comprised of 19,662 CHD cases and 8,992 controls with 96 clinical features, which were then used to train, validate, and evaluate the prediction models.

Overall, all individuals were included in the total clinical dataset from China and had a mean age of 62.3 years (standard deviation of 13.4 years). Male patients accounted for 63.3% of the study cohort (Table 1). The proportion of male patients was slightly higher in the CHD group (68.3%) than that in the control group (52.5%). All individuals in this study were Chinese, with 63.2% from Han and 33.6% from Zhuang ethics groups. Han and Zhuang ethics groups constituted the majority of the population in Guangxi Province, which explains the cohort composition of this study. Approximately 54.2% of the patients reported smoking or had a smoking history, and 57.4% of the patients reported excessively drinking or had an excessive drinking history. With respect to comorbidities, 29.8% of the patients were diagnosed with pre-diabetes or diabetes mellitus, 60.9% with hypertension, 16.5% with hypercholesterolemia, 12.9% with fatty liver disease, and 10.6% with hyperuricemia (Table 1). In terms of medicine intake, 78.8% of the CHD cohort and 63.4% in the controls were reported using hypertension depressor. The typical lab measurements are listed in Table 1, including low-density lipoprotein (LDL) cholesterol, HDL cholesterol, total cholesterol, apolipoprotein AI, triglycerides, glucose, hemoglobin and lactate dehydrogenase. The *t*-test results indicated no significant differences in lab measurements among the whole cohort, patients with CHD, and controls.

### 3.2 Prediction of on-site CHD risk

**Fig 2:**
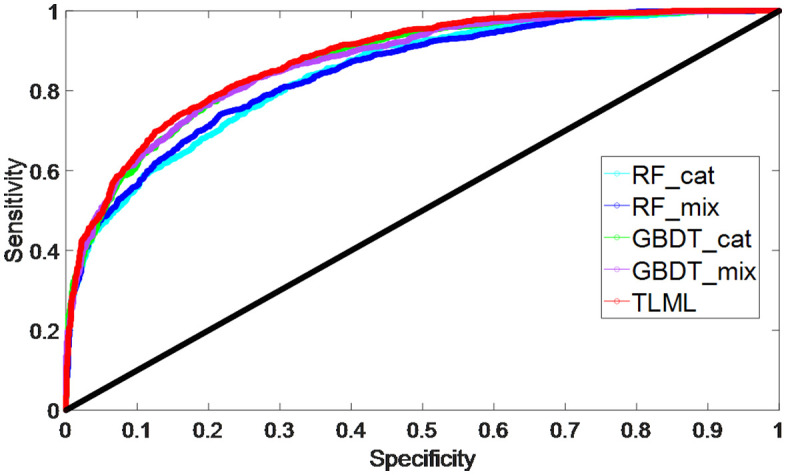
The receiver-operating characteristics curves for CHD prediction models. TLML: two-layer machine learning model; cat: model trained with all-categorical data; mix: model trained with mixture of numerical and categorical data.

The RF_cat model predicted CHD with an AUROC of 0.84 (95% CI 0.84–0.85), an accuracy of 0.73 (95% CI 0.72–0.73), a sensitivity of 0.91 (95% CI 0.91–0.92), and a specificity of 0.54 (95% CI 0.52–0.55) (Fig 2), indicating that the RF_cat model was aggressive in predicting CHD cases resulting in many false positives. A PPV of 0.67 (95% CI 0.66–0.67) and an NPV of 0.86 (95% CI 0.86–0.87) were achieved with RF_cat (Table 2), indicating that RF_cat showed better performance predicting negative samples than positive samples. An F-score of 0.70 was obtained by with RF_cat model, indicating that RF_cat could achieve a good CHD risk prediction.

**Table 2:** The prediction performance from different models.

Models	AUROC	accuracy	sensitivity	specificity	PPV	NPV	F-score
RF_cat 95% CI	0.84 0.84-0.85	0.73 0.72-0.73	0.91 0.91-0.92	0.54 0.52-0.55	0.67 0.66-0.67	0.86 0.86-0.87	0.70 0.69-0.71
RF_mix 95% CI	0.84 0.84-0.85	0.73 0.73-0.74	0.89 0.89-0.90	0.58 0.57-0.58	0.68 0.67-0.68	0.84 0.84-0.85	0.72 0.71-0.72
GBDT_cat 95% CI	0.87 0.87-0.87	0.78 0.77-0.78	0.79 0.78-0.79	0.78 0.77-0.78	0.78 0.77-0.78	0.78 0.78-0.78	0.78 0.78-0.78
GBDT_mix 95% CI	0.87 0.87-0.88	0.79 0.78-0.79	0.79 0.78-0.79	0.78 0.78-0.79	0.78 0.78-0.79	0.79 0.78-0.79	0.79 0.79-0.79
TLML 95% CI	0.88 0.88-0.88	0.79 0.79-0.80	0.79 0.79-0.80	0.79 0.79-0.79	0.79 0.79-0.79	0.79 0.79-0.79	0.79 0.79-0.79
RCM 95% CI	0.80 0.80-0.81	0.73 0.72-0.73	0.74 0.74-0.75	0.71 0.70-0.71	0.71 0.71-0.72	0.74 0.73-0.74	0.73 0.72-0.73

AUROC: area under receiver operating characteristic curve. PPV: positive predictive value.

NPV: negative predictive value. RF: random forest. cat: model trained with all-categorical data.

mix: model trained with mixture of numerical and categorical data. CI: confidence interval.

GBDT: gradient boosting decision tree. RCM: reduced complexity model.

TLML: two-layer machine learning model.

The GBDT_cat predicted CHD with an AUROC of 0.87 (95% CI 0.87–0.87), an accuracy of 0.78 (95% CI 0.77–0.78), a sensitivity of 0.79 (95% CI 0.78–0.79) and a specificity of 0.78 (95% CI 0.77–0.78) (Fig 2). A PPV was evaluated as 0.78 (95% CI 0.77–0.78), and an NPV as 0.78 (95% CI 0.78–0.78). An F-score of 0.78 (95% CI 0.78–0.78) was achieved with the GBDT_cat. Using the categorical dataset, the prediction of CHD risk by GBDT_cat was slightly better than that by RF_cat model by 2.5% based on AUROC, 5.0% based on accuracy and 7.7% based on F-score (*p* value < 0.05); however, in terms of sensitivity and NPV, the RF_cat model was 13.5% and 8.4% better than those predicted by GBDT_cat respectively (*p* value < 0.05). The difference in the prediction measurements suggested that various machine learning models can result in different prediction performances.

When training machine learning models with categorical/continuous clinical data, the RF_mix and GBDT_mix showed different performances compared with those trained with all-categorical data. The RF_mix model predicted CHD with an AUROC of 0.84 (95% CI 0.84–0.85) and an accuracy of 0.73 (95% CI 0.73–0.74) (Fig 2). A sensitivity of 0.89 (95% CI 0.89–0.90), a specificity of 0.58 (95% CI 0.57–0.58), a PPV of 0.68, an NPV of 0.84, and an F-score of 0.72 (95% CI 0.84–0.85) were obtained from RF_mix (Table 2). The GBDT_mix model predicted CHD with an AUROC of 0.87 (95% CI 0.87–0.88) (Fig 2), an accuracy of 0.79 (95% CI 0.78–0.79) (Table 2). A F-score of 0.79 with 95% CI of 0.78–0.79 was obtained from the GBDT_mix. The RF_mix and RF_cat models gave similar AUROC but not accuracy and F-score (*p* value<0.02). Similar trends were also found in the result of GBDT models.

Combining the merits of the four trained models, the TLML can generate a better prediction for CHD, with an AUROC of 0.88 (95% CI 0.88–0.88), an accuracy of 0.79 (95% CI 0.79–0.80) and an F-score of 0.79 (95% CI 0.79–0.79) (Fig 2). The TLML model was better at predicting CHD based on the AUROC, accuracy and F-score (*p* value<0.01) than the single machine learning model, indicating adding a second layer of NN model could further improve CHD prediction. A sensitivity of 0.79, specificity of 0.79, a PPV of 0.79, and NPV of 0.79 were obtained with TLML (Table 2). Compared with RF_cat and RF_mix, there was a decrease in sensitivity but an increase in specificity, indicating the TLML corrected some of false positive prediction made by RF_cat and RF_mix. The predictive performance of the TLML was also compared between the male and female subgroups in the validation dataset, resulting in no significant difference (*p* value>0.1). This indicated that the TLML did not have bias predicting CHD risk for man or woman. Similarly, the difference in CHD prediction between the Han and Zhuang ethnics were not found to be significant (*p* value>0.1).

**Fig 3:**
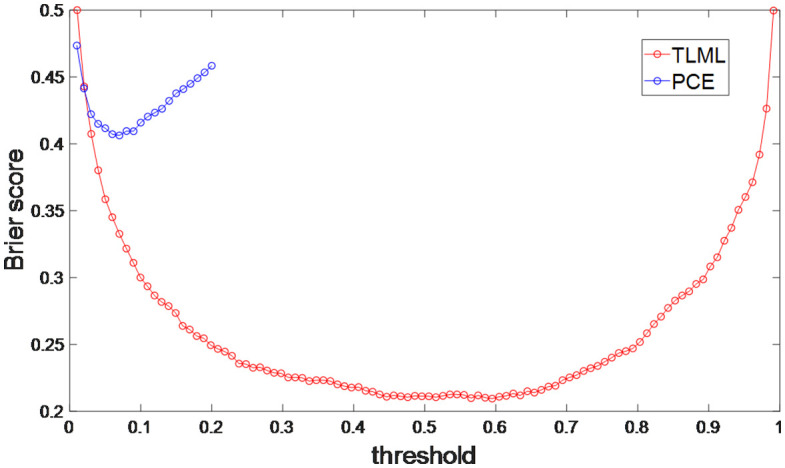
The Brier scores of the prediction from PCEs and TLML with different thresholds. TLML: two-layer machine learning model, PCEs: pooled cohort equations.

Because a good on-site prediction from TLML was obtained, a TLML risk score was worth developing to provide a quantitative indicator for CHD diagnostics. The prediction scores from TLML were used as quantitative risk indicators for on-site CHD and ranged from 0 to 1. The higher the risk score, the higher the posterior probability of CHD. The Brier scores optimization of the TLML showed that the lowest Brier score (0.2) was obtained with a threshold of 0.5 (95% CI of 0.47–0.53) (Fig 3). This threshold can be used as a reference to classify patients into high CHD risk and low CHD risk groups.

The generalizability of the TLML model was further analyzed by justifying the sample size and overfitting risk. The study included 20 iterations of training and validation of models to minimize the effect of sampling to the predictive performance, where 95% confidence interval was narrow (Table 2) of each performance metrics (≤0.02), indicating that consistent predictive performances of the TLML models among all iterations. The narrow confidence interval also indicated that the sample size was large enough for reliable model development, which was also supported by the learning curve result obtained by varying training and validation cohort size (S1 Fig). The AUROC of the TLML model plateaued at the 100% dataset inclusion. The overfitting risk was mitigated by including a 10-fold cross-validation during training. In addition, the predictive performance of the training and validation dataset was compared under different sample sizes (S2 Table). Similar predictive accuracy was similar (*p* value>0.1), indicating the overfitting of the TLML model was small.

The 20 most important clinical features (S1 Table) for CHD prediction were ranked by their contribution. The top 12 were age, diabetes, antihypertensive medication use, total bilirubin, hypertension, obstructive sleep apnea-hypopnea syndrome (OSAHS), red blood cell (RBC) count, hemoglobin, cystatin C, retinol-binding protein (RBP), sex, and LDL cholesterol levels. All the clinical features mentioned above were found relevant to CHD. The link between some of the 12 clinical features and CHD has been accepted, whereas the link between others and CHD has not been studied well.

Age, diabetes, taking depressor, hypertension and LDL cholesterol level were commonly used to predict the CHD risks. PCEs [14] assess the 10-year ASCVD risk in asymptomatic individuals, which involves the input of age, sex, race/ethnicity, diabetes, smoking, SBP, antihypertensive use, total cholesterol levels, and HDL cholesterol levels. The most relevant predictors presented in this paper are consistent with those in PCEs, except that race/ethnicity and smoking were considered less important in this study compared with the rest. Race/ethnicity is less important may be due to our samples were all from China, which the Han and Zhuang ethnic groups are much closer than the relationship between, for example, White and African American in PCEs. In a Chinese-oriented ASCVD prediction model (China-Par) [8], risk factors, such as sex, age, region, waist circumference, total cholesterol level, HDL cholesterol level, blood pressure, antihypertensive medication use, diabetes, smoking, and family history of ASCVD, are included in the prediction equations, but race/ethnicity is not included, which supports the results of this study that race/ethnicity is less important in CHD prediction in the Chinese population.

Except for the clinical features mentioned in other prediction models, clinical features such as total bilirubin, OSAHS, RBC count, hemoglobin, cystatin C, and RBP were less frequently mentioned in CHD prediction models. Total bilirubin level has been suggested to have a protective effect against CHD. A study of dose-response meta-analysis supported the U-shaped dose-response relationship between CHD and total bilirubin [15], consistent with previous studies [16,17]. OSAHS may be a good indicator of CHD or ASCVD because of its association with obesity and metabolic changes. The link between OSAHS and ASCVD has been reviewed [18], showing that addressing OSAHS relevant obesity could be an effective strategy for reducing cardiovascular risk. Another study pointed out that patients with OSAHS complicated by CHD showed a significant increase in inflammatory factors, glycolipid metabolism, obesity rate, and homeostasis model assessment of insulin resistance compared with those in the OSAHS group, indicating that OSAHS could be an indicator of CHD [19]. RBC disorder was believed to have a pathophysiological link with ASCVD, and a previous study recommended including RBC count and RBC distribution width in ASCVD risk score calculation [20]. Hemoglobin levels have also been reported to be associated with the CHD or ASCVD risk. For example, a previous study reported that an increased CHD risk was significant when the hemoglobin level exceeded 17.0 g/dL [21]. Another study of a Japanese cohort without diabetes showed that both low and high hemoglobin A1c levels were associated with a higher ASCVD risk [22]. Cystatin C, an endogenous marker of kidney function, was reported to be able to independently predict major cardiovascular events over 6 years and long-term cardiovascular mortality at 16-year follow-up in patients with CHD [23], indicating that cystatin C is associated with CHD risks. The feasibility of cardiovascular events prediction with high cystatin C concentrations was also supported by another study on ambulatory patients with CHD [24]. However, some studies have denied the correlation between cystatin C and CHD, making this relationship controversial. For example, Mendelian randomization analyses did not support a causal role for cystatin C in the etiology of ASCVD [25]. RBP plays a role in the pathophysiology of CHD through their involvement in the progression of inflammatory mechanisms [26], but the link between CHD and RBP is also vague according to previous studies. Low-quality correlation was found between CHD and circulating RBP4 levels, because a significant association was only found under the random-effect model in their study but not others. However, full-length and total RBP4 levels have been reported associated with an increased CHD risk in a time-dependent manner [27]. Other studies have reported that the high level of RBP4 had association with increased risk in adverse cardiovascular events in patients with CHD [28] and increased ASCVD risk interacting with hypertension [29].

### 3.3 Prediction of CHD risk by PCEs

**Fig 4:**
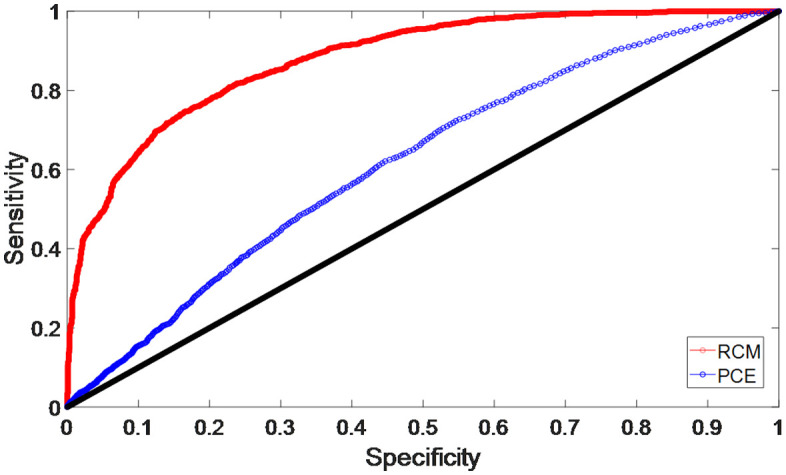
The receiver-operating characteristics curves for PCEs and RCM. TLML: two-layer machine learning model; RCM: reduced complexity model.

The patients with CHD accounted for 50.3% of the cohort for PCEs prediction. Individuals with risk >7.5% were considered to have high CHD risk, according to the instructions. The PCEs predicted CHD with an accuracy of 0.59, a sensitivity of 0.58 and a specificity of 0.60. The NPV was achieved as 0.67, and the PPV was 0.51. The AUROC and F-scores of the PCEs prediction were shown to be 0.59 and 0.62, respectively (Fig 4), which are 29% and 17% lower than those obtained with the TLML. An accuracy of only 0.59 indicated that the PCEs could only provide a limited prediction of on-site CHD.

The inaccuracy of the PCEs for CHD prediction in this study may be attributed to several reasons. First, the PCEs were designed for 10-year CHD risk prediction; therefore, the on-site CHD risk prediction provided by the PCEs could be inaccurate. Second, the prediction of on-site CHD may require more clinical features than those included in the PCEs’ input. The TLML model contained 96 clinical features, whereas the PCEs only required 12. Finally, the threshold selected for on-site CHD risk classification may not be optimized when using the PCEs. Therefore, the threshold for CHD risk classification was optimized by obtaining a minimum Brier score, which showed that the best prediction was achieved with a risk threshold of 7.0% (Brier score = 0.41) (Fig 3). The optimized threshold was close to the original threshold (7.5%), illustrating that the risk threshold selection was not the predominant factors influencing the prediction performance using the PCEs.

### 3.4 Prediction of CHD with the RCM

Compared with the TLML, the RCM remained good prediction performance, with number of predictors reduced to 20. The RCM predicted CHD with an AUROC of 0.80 (95% CI 0.80–0.81), an accuracy of 0.73 (95% CI 0.72–0.73), a sensitivity of 0.74 (95% CI 0.74–0.75), a specificity of 0.71 (95% CI 0.70–0.71), a PPV of 0.71 (95% CI 0.71–0.72) and an NPV of 0.74 (95% CI 0.73–0.74) (Fig 4). The F-score of the RCM is 0.73 (95% CI 0.72–0.73). Although the AUROC, F-score and prediction accuracy of the RCM were remarkably lower than those of the TLML (*p* value<0.01), the RCM exhibited significantly better and reliable prediction of on-site CHD than the PCEs (baseline). The RCM provided 15% better accuracy than that with the PCEs using a comparable number of inputs, indicating that the RCM used different mechanisms to predict on-site CHD than the PCEs.

The RCM requires fewer predictors, making it a more practical alternative to TLML, only sacrificing small prediction accuracy. The predictors included in the RCM shared some common clinical features with the PCEs, but required additional features such as creatinine and uric acid, etc. The inclusion of more clinical features can provide additional information for the model prediction. Meanwhile, the 20 predictors included in the RCM were all from vital traits, lab measurements, etc. during admission to the hospital, so applicability could be ensured.

### 3.5 Outlook and study limitation

The prediction of on-site CHD using TLML and RCM presented in this study is quite meaningful as a quantitative CHD risk assessment tool that can provide an auxiliary measurement for patients before coronary angiography. The early diagnostics of CHD with TLML can contribute to interventions for coronary artery lesions, which can decrease the incidence of acute myocardial infarction, death caused by ASCVD, and sudden cardiac death. The application of TLML can help reduce the probability of unnecessary coronary angiography, which is both costly, time-consuming, and traumatic. The RCM can generate a good level of prediction with only 20 input clinical features, indicating that it has a better chance of being applied in practice. However, it should be highlighted that the TLML or the RCM can be applied in practice in the future only with the acceptance of the patients and physicians. Both physicians and patients require training and promotion.

This study had some limitations. First, the clinical data used in this study was limited by the total sample size. The limitations of the training samples can lead to overfitting, which reduces the generalizability of the prediction models presented in this study. This limitation of sample size was mitigated by applying 20 iterations and sample size influence analysis, the results of which indicated little sample size influence. Second, this study was retrospective in its use of clinical data and limited to only one hospital. Although regional and ethnic variations (24 out of 34 provinces in China and 3 different ethnic groups) were included in samples, the generalizability of the presented prediction models may still be weakened due to the limited availability of the dataset. A broader population should be studied in the future using the prediction models presented in this study. Third, the information excluded from the prediction models, such as clinical features discarded because of low data integrity and features not recorded in the clinical data (e.g., nutrients and physical activity, etc.) may also be informative for on-site CHD risk prediction. Fourth, the benchmark prediction model was limited to the PCEs in this study because of its accessibility. Other models, such as China-PAR, can be compared with machine learning models for on-site CHD prediction in later studies. Finally, the TLML model is a black box, which limits its direct interpretability. Although the models demonstrated high predictive accuracy, further efforts are needed to improve its transparency. Future studies should explore the use of interpretability techniques to increase model transparency.

## Conclusions

CHD is one of the noninfectious chronic diseases with the highest morbidity and mortality, and tends to be more prevalent in the future. Unfortunately, current diagnosis is based on the result of coronary angiography, which is traumatic and expensive. On-site CHD risk prediction before committing coronary angiography would be meaningful as an auxiliary diagnosis measurement using the clinical data during admission to the hospital. A TLML is developed to predict CHD risk with the clinical data from the Second Affiliated Hospital of Guangxi Medical University. The model can provide a good CHD risk prediction compared with widely used PCEs, and potentially serve as a quantitative on-site CHD risk evaluation tool. RCM is also developed to increase model practicality but remains a good prediction with only top 20 most relevant clinical features, which are all found to have an association with CHD. The acceptance of the patients and physicians, training and promotions will be required before application in the future.

## Supporting information

S1 Table The top 20 most important features among all coronary heart disease predictors. Higher measures indicate more importance.
(PDF)

S2 Table The comparison of the predictive performance of TLML model between the training and validation cohort with different data inclusion, where 100% of data inclusion was the same as the original study design. (PDF)

S1 Fig The change of the area under receiver operating characteristic curve for all the models presented with the increasing percentage of cohort included in training and validation. The number of samples for the 100% of dataset inclusion was consistent with that for prediction model development in this study. TLML: two-layer machine learning model; cat: model trained with all-categorical data; mix: model trained with mixture of numerical and categorical data.
(PDF)
