# Supplementary material for: Development and validation of a machine learning model for on-site prediction of coronary heart disease in high-risk adults using clinical data
Source: PLoS One. 2025 Nov 13;20(11):e0334881. doi: 10.1371/journal.pone.0334881 (PMC12614581; doi:10.1371/journal.pone.0334881)
Supplement: S1 Table — Higher measures indicate more importance. (PDF) [file pone.0334881.s001.pdf]

S1 Table The top 20 most important features among all coronary heart disease predictors. Higher measure indicates more importance.

Rank	Feature	Measure
1	Age	0.86
2	Diabetes	0.54
3	Antihypertensive use	0.46
4	Total bilirubin	0.39
5	Hypertension	0.38
6	Obstructive sleep apnea-hypopnea syndrome	0.37
7	Red cell count	0.35
8	Hemoglobin	0.32
9	Cystatin C	0.32
10	Retinol-binding protein	0.30
11	Gender	0.30
12	Low-density lipoprotein cholesterol level	0.29
13	Indirect bilirubin	0.29
14	Hematocrit	0.28
15	Direct bilirubin	0.28
16	Uric acid	0.26
17	Glutamyl transpeptidase	0.25
18	Creatinine	0.25
19	High-density lipoprotein cholesterol	0.25
20	Direct bilirubin/Total bilirubin	0.25
