# Supplementary material for: Development and validation of a machine learning model for on-site prediction of coronary heart disease in high-risk adults using clinical data
Source: PLoS One. 2025 Nov 13;20(11):e0334881. doi: 10.1371/journal.pone.0334881 (PMC12614581; doi:10.1371/journal.pone.0334881)
Supplement: S2 Table — (PDF) [file pone.0334881.s002.pdf]

S2 Table The comparison of the predictive performance of TLML model between the training and validation cohort with different data inclusion, where 100% of data inclusion was the same with the original study design.

Data inclusion %	Accuracy	
	Training dataset	Validation dataset
30	0.97±0.1	0.97±0.1
40	0.93±0.1	0.93±0.1
50	0.89±0.1	0.90±0.1
60	0.88±0.1	0.88±0.1
70	0.87±0.1	0.87±0.1
80	0.86±0.1	0.86±0.1
90	0.85±0.1	0.85±0.1
100	0.85±0.1	0.85±0.1

TLML: two-layer machine learning model.
