# Supplementary material for: Development and validation of a machine learning model for on-site prediction of coronary heart disease in high-risk adults using clinical data
Source: PLoS One. 2025 Nov 13;20(11):e0334881. doi: 10.1371/journal.pone.0334881 (PMC12614581; doi:10.1371/journal.pone.0334881)
Supplement: S1 Fig — The number of samples for the 100% of dataset inclusion was consistent with that for prediction model development in this study. TLML: two-layer machine learning model; cat: model trained with all-categorical data; mix: model trained with mixture of numerical and categorical data. (PDF) [file pone.0334881.s003.pdf]

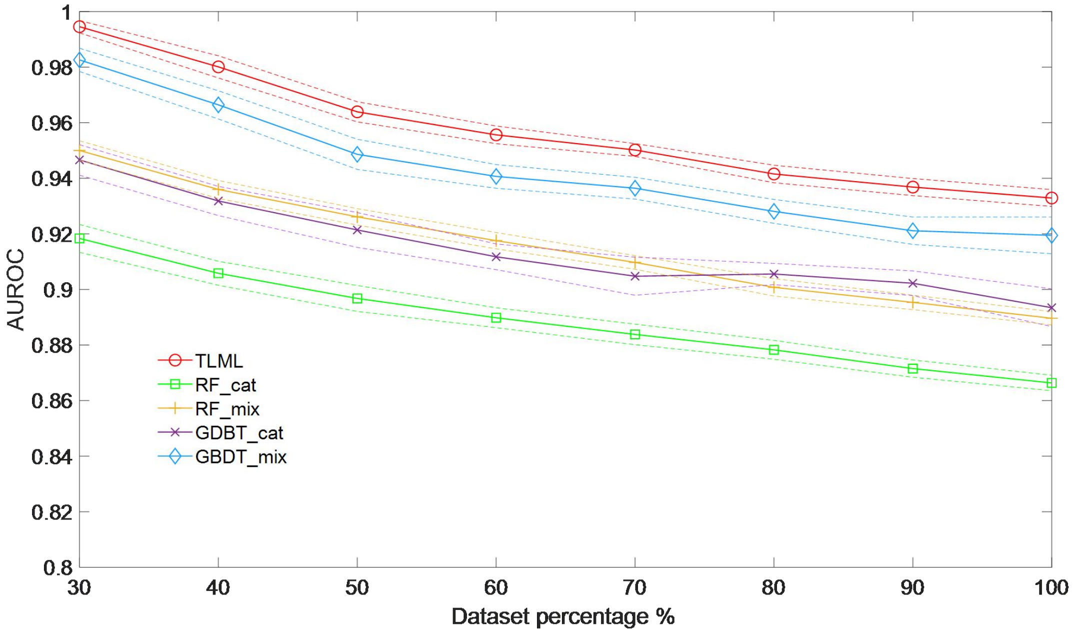

S1 Fig The change of the area under receiver operating characteristic curve for all the models presented with the increasing percentage of cohort included in training and validation. The number of samples for the 100% of dataset inclusion was consistent with that for prediction model development in this study. TLML: two-layer machine learning model; cat: model trained with all-categorical data; mix: model trained with mixture of numerical and categorical data.
